# Supplementary material for: Autosomal Dominant Alzheimer’s Disease Mutations in Human Microglia Are Not Sufficient to Trigger Amyloid Pathology in WT Mice but Might Affect Pathology in 5XFAD Mice
Source: Int J Mol Sci. 2024 Feb 22;25(5):2565. doi: 10.3390/ijms25052565 (PMC10932392; doi:10.3390/ijms25052565)
Supplement: Supplementary file 1 [file ijms-25-02565-s001.zip › Supplementary figures.pdf]

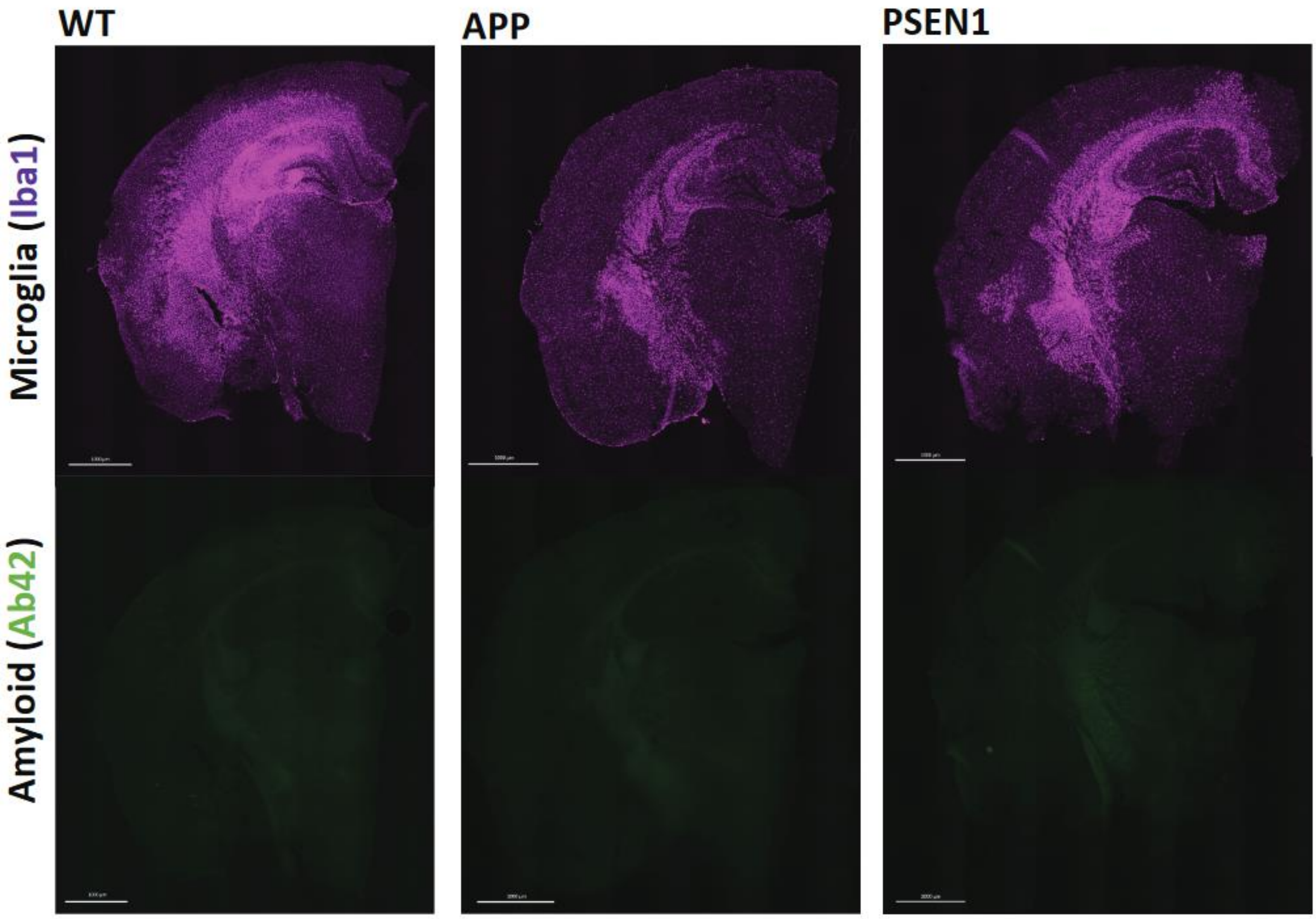

Figure S1. Human induced microglia from *APP* and *PSEN1* mutation carriers do not trigger amyloid pathology when xenotransplanted into the mouse brain. A) Representative images of 6 month-old hCSF1 WT mice xenotransplanted with human WT or ADAD *APP* or *PSEN1* mutation-carrying) microglia, stained with IBA1 (microglia marker) or Aβ42 (amyloid marker). Scale bars = 1000 μm.

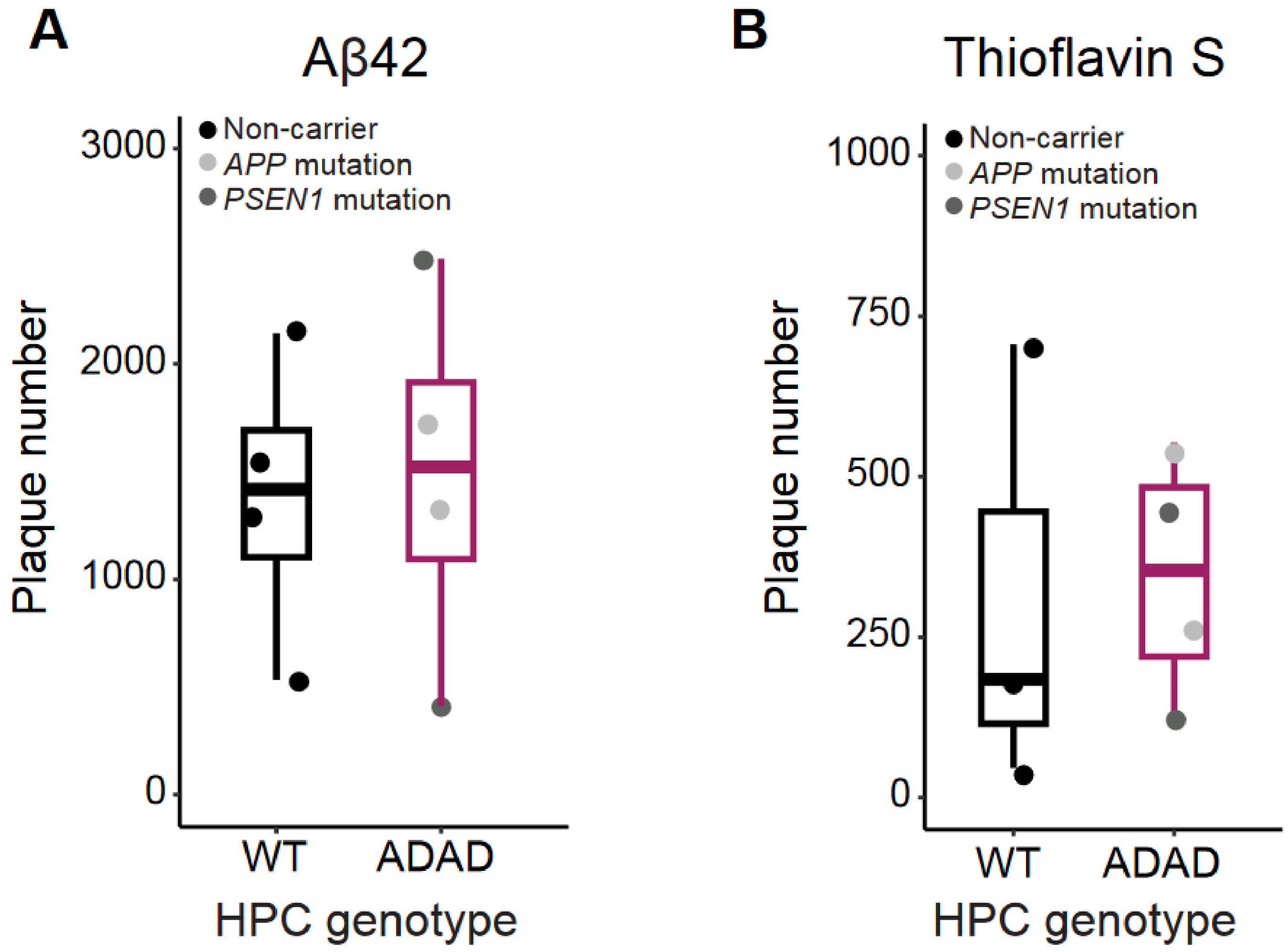

Figure S2. Amyloid plaque number quantified by Aβ42 (A) or ThioS (B) stainings in 6-month-old hCSF1 WT and 5XFAD mice xenotransplanted with WT and ADAD HPCs. Each dot represents one donor line (N=3-4 WT + N=4 ADAD donor lines, 1-5 xenotransplanted mice per donor line, 4-7 brain sections analyzed per mouse). For Aβ42: ADAD – WT [95% confidence interval] = -5.5 [-777, 766] μm^2, P-value = 0.989); for ThioS: ADAD – WT [95% confidence interval] = 29.3 [-263, 321] μm^2, P-value = 0.848).

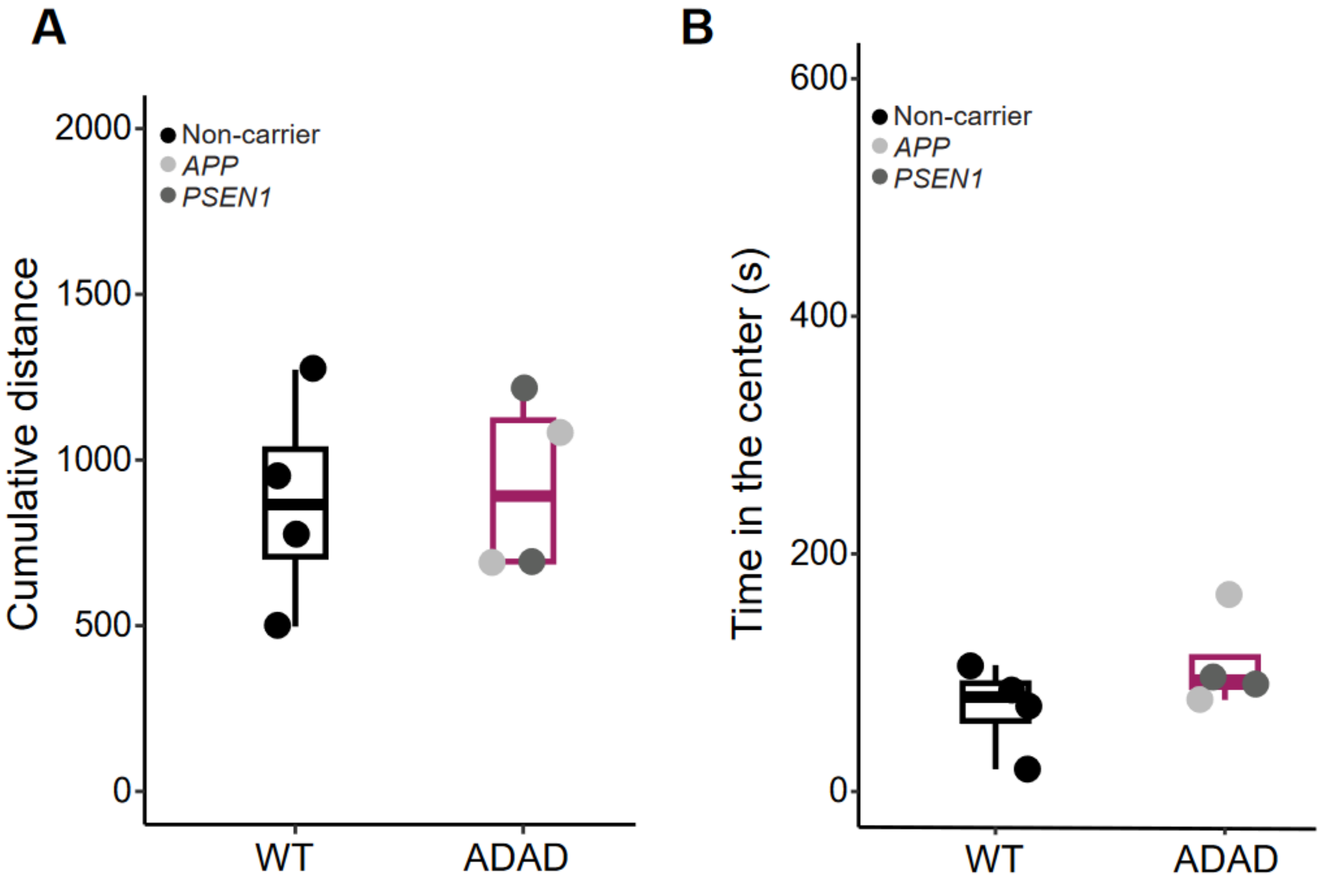

Figure S3. Open field behavioral test. Cumulative distance (A) and duration in the center of the field (B) in 6-month-old hCSF1 5XFAD mice xenotransplanted with WT and ADAD HPCs. Each dot represents one donor line (N=3-4 WT + 4 ADAD donor lines, 2-6 xenotransplanted mice per donor line).
